# Limits and pitfalls of indirect revascularization in moyamoya disease and syndrome

**DOI:** 10.1007/s10143-020-01393-1

**Published:** 2020-09-21

**Authors:** Pietro Fiaschi, Marcello Scala, Gianluca Piatelli, Domenico Tortora, Francesca Secci, Armando Cama, Marco Pavanello

**Affiliations:** 1 Department of Neurosurgery, IRCCS Ospedale Policlinico San Martino, Genoa, Italy; 2 Department of Neurosciences, Rehabilitation, Ophthalmology, Genetics, Maternal and Child Health, University of Genoa, Genoa, Italy; 3 Department of Neurosurgery, IRCCS Istituto Giannina Gaslini, Via Gerolamo Gaslini, Genoa, Italy; 4 Neuroradiology Unit, IRCCS Istituto Giannina Gaslini, Genova, Italy

**Keywords:** Moyamoya disease, Moyamoya syndrome, Indirect revascularization, EDAS, Bypass

## Abstract

Moyamoya vasculopathy is a rare chronic cerebrovascular disorder characterized by the stenosis of the terminal branches of the internal carotid arteries and the proximal tracts of anterior and middle cerebral arteries. Although surgical revascularization does not significantly change the underlying pathogenic mechanisms, it plays a pivotal role in the management of affected individuals, allowing to decrease the risk of ischemic and hemorrhagic complications. Surgical approaches may be direct (extracranial-intracranial bypass), indirect, or a combination of the two. Several indirect techniques classifiable according to the tissue (muscle, periosteum, galea, dura mater, and extracranial tissues) or vessel (artery) used as a source of blood supply are currently available. In this study, we reviewed the pertinent literature and analyzed the advantages, disadvantages, and pitfalls of the most relevant indirect revascularization techniques. We discussed the technical aspects and the therapeutical implications of each procedure, providing a current state-of-the-art overview on the limits and pitfalls of indirect revascularization in the treatment of moyamoya vasculopathy.

## Introduction

Moyamoya is a chronic cerebrovascular disorder consisting in the bilateral stenosis or occlusion of the terminal portion of the internal carotid arteries and the proximal tracts of the anterior and middle cerebral arteries [1–3]. This condition can be classified into pure moyamoya disease (MMD), when no associated risk factor is identifiable, and moyamoya syndrome (MMS) or quasi-MMD, when the vasculopathy develops in association with clear risk factors [2, 4, 5]. Surgical treatment remains the cornerstone in patient management in order to reduce the risk ischemic or hemorrhagic sequelae [4, 6]. However, it has no significant impact on the pathogenic mechanisms underlying moyamoya vasculopathy [6].

Surgical techniques can be categorized as direct, indirect, or a combination of the two [7]. Extracranial-intracranial bypass (e.g., STA-MCA anastomosis) is generally the standard direct surgical procedure [8]. Indirect revascularization techniques employ a variety of tissues as a source of blood supply to induce angiogenesis [6, 9]. Combined revascularization refers to the association of the direct and indirect revascularization techniques [6]. The several existing variations of indirect revascularization can be classified according to the donor vessel or tissues employed.

To date, the available techniques have been only partially described in terms of efficacy and safety in separate case series, but a direct and comprehensive overview is still lacking. The aim of this study is to analyze advantages, disadvantages, and pitfalls of indirect revascularization techniques. We conducted a detailed literature search and discussed the peculiar technical and therapeutical aspects of each approach, focusing on the most relevant recent advancements in this field of moyamoya surgical treatment.

## Methods

A comprehensive literature search was performed using the PubMed database. We searched for the terms “moyamoya,” “indirect revascularization,” “bypass,” “synangiosis,” and “stroke.” All the studies between 1977 and 2020 assessing the technical aspects and clinic-radiological results of indirect revascularization techniques in the treatment of moyamoya vasculopathy were reviewed. We included clinical case reports and excluded non-English articles and single abstracts.

### Classification of procedures according to donor tissue

Every indirect revascularization procedure is applicable in both adult and pediatric patients. However, the choice of the most appropriate approach in each case must take into account the size of vessels, the availability of the donor tissue, the possibility of developing an effective collateral circulation, the patient’s age, the severity of the neurological deficits, and the degree of disability at the disease onset. The coexistence of associated comorbidities (especially in MMS) also influences this decisional process.

### Donor tissue: artery

#### Encephaloduroarteriosynangiosis/occipital synangiosis

**Fig. 1:**
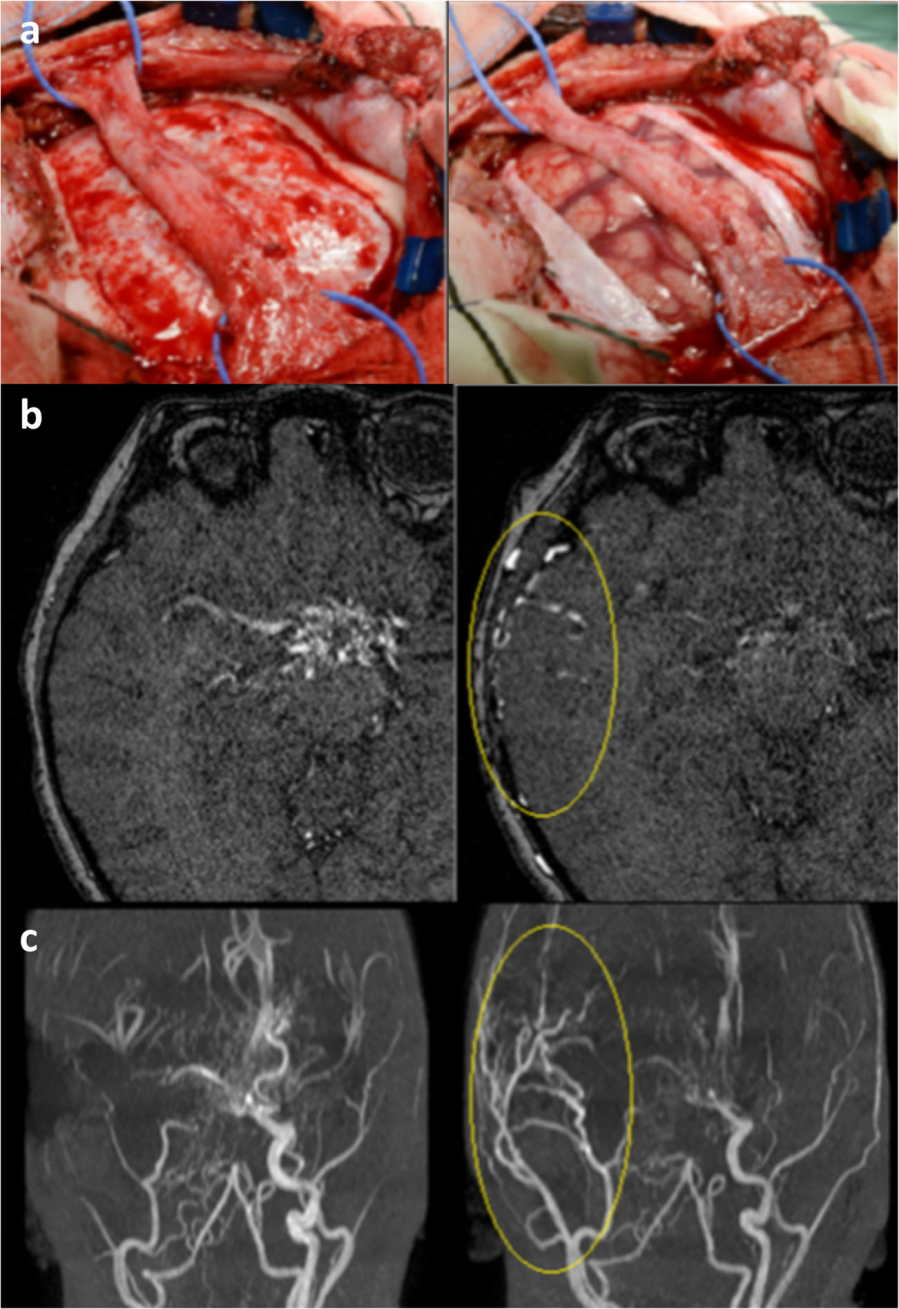
Illustration of EDAS procedure. **a** Surgical procedure of encephaloduroarteriosynangiosis (EDAS) with the arterial flap containing the parietal branch of the superficial temporal artery (STA) and surrounding galea tissue for an extension of 10 cm directly in contact with the cerebral cortex. The extremities of the bridge gently degrade so as not to create kinking of artery. Preoperative (**b**) and 1-year postoperative (**c**) magnetic resonance angiography (MRA) study shows the extensive collateralization in territory ACM (yellow circle)

Encephaloduroarteriosynangiosis (EDAS) is the most popular indirect technique for surgical revascularization (Fig. 1). Each branch of the superficial temporal artery (STA) can be used in relation to the territory involved by the vasculopathy. In particular, the frontal and parietal branches can be used if the middle cerebral artery (MCA) or the anterior cerebral artery (ACA) are affected, respectively. Technically, after being separated from underlying periosteum and temporalis fascia, STA is directly sutured to dural edges in a watertight fashion [10].

Some criticisms of this technique have been reported in the literature, the most relevant of which concerns the poor collateralization achieved. In fact, EDAS alone has been judged to be insufficient for the vascular supplying of the whole cortical surface, especially if the ACA territory is involved and the potential revascularization area is confined to the craniotomy field. As a matter of fact, in most patients, a satisfactory blood flow is gained only in a limited area surrounding the surgical field [11, 12]. Furthermore, postoperative single-photon emission computed tomography (SPECT) studies demonstrated that EDAS did not normalize the regional CBF distribution. This finding was particularly evident in the frontal lobe, because of its limited surgical exposure [12]. As EDAS insufficiently prevents ischemia in the frontal and occipital lobes, Tenjin et al. proposed a multiple-EDAS technique based on the use of the frontal branch of STA and the occipital artery as an effective and safe method to prevent ischemia in such territories [13].

EDAS limitation has encouraged many centers to perform additional techniques in refractory cases. Kim et al. showed that EDAS combined with bifrontal EGPS significantly improved ACA-related symptoms (81% vs 40%) and the rate of revascularization as compared to EDAS alone. However, the incidence of postoperative infarction was not significantly different between the two studied cohorts and the final clinical outcomes were not associated with the surgical modality [14]. Park et al. introduced a modified EDAS with bifrontal encephalogaleoperiostealsynangiosis (EGPS) [15]. SPECT scans showed improved vascular reserve capacity in both the ACA and MCA territories in 14 out of the 17 patients with MMD studied (82.4%) [15]. The technique entails the insertion of a dural fold into the interhemispheric fissure, which provides contact between the outer layer of the dura mater and the interhemispheric frontal lobe surface. A second fold covering the paramedian anterior surface of the frontal lobe can be made with a galeo-periosteal flap, which should be as wide as possible. Particular attention should be paid during the insertion of the flap into the interhemispheric surface to avoid the damage of draining veins [15]. The main disadvantages of this approach are the procedure length and the risks of postoperative ischemia and infection [15].

In case of failure of the EDAS, the conversion to a direct technique is often conditioned by the ineligible diameter of cortical arteries (< 0.6 mm). Indeed, in spite of the revascularization defect, EDAS results in the adhesion of the donor tissue to the cortical surface. Accordingly, the integrity of the temporal branch artery has to be preserved. Another possibility is represented by the conversion into a rescue technique. Matsushima et al. described a better vascular collateralization achieved with the addition of encephalomyosynangiosis (EMS) or encephalomyoarteriosynangiosis (EMAS) in 3 patients in which EDAS had failed [16]. Touho et al. performed omental transplantation using a branch of the STA which had been previously used in an EDAS procedure. Good clinical and angiographic results were achieved in all 5 patients [17].

In children, skin necrosis can occur at the site of the STA dissection. To avoid this eventuality, the subcutaneous tissue including a scalp artery and a relevant vein should be as thin as possible [18]. In general, skin necrosis may result difficult to manage and represent a life-threatening problem when the infection involves the non-vascularized skull undergoing surgery. Chung Y et al. proposed a simple “layering” technique of “In-to-Out” dissection method that can protect against bacterial contamination and reduce postoperative surgical wound problems [19]. In this procedure, the sealing of the galea aponeurotica (including fibrous septa) and loose areolar tissues (including the periosteal layer) is an essential factor to decrease the risk of scalp wound infection.

#### Pial synangiosis

This procedure consists in the suturing of the STA to the underlying pia. Technically, the intact donor artery is sutured by its galeal cuff directly to the pial surface. Accordingly, it is necessary to reduce to the minimum the gap from the cortex. In a study by Golby et al, postoperative digital subtraction angiography showed Matsushima A-B revascularization in 89.5% of the hemispheres of patients treated with pial synangiosis in all age groups [7]. Jackson et al. further showed that pial synangiosis is an effective and durable method to reduce the risk of stroke in patients under 2 years of age [20].

The occipital synangiosis technique is similar to EDAS, but it is limited to the territory of the posterior cerebral artery (PCA). Kimiwada et al. observed progressive PCA stenosis in 19.4% of pediatric patients younger than 16 years after anterior revascularization [21]. In particular, the presence of cerebral infarction and the younger age at the time of the initial diagnosis have been identified as relevant risk factors for the development of progressive PCA stenosis following an anterior revascularization procedure. Lee et al. further reported improved symptoms and radiological findings in 12.2% of 335 pediatric patients with MMD treated with indirect posterior revascularization surgery, including EDAS and multiple burr holes [22]. Of note, this procedure is only feasible when recipient arteries are available for the anastomosis to the brain surface. An important complication to mention is the graft dissection, which is mainly caused by the tortuous route of the graft and its deep location in the occipital muscle [23].

### Donor tissue: muscle

#### EMS-EDMS-EDAMS and ribbon EDAMS/EDMAPS

EMS, first introduced by Karasawa in 1977, should be considered an intermediate surgical option consisting in the dissection of the temporalis muscle and its subsequent placement on the brain surface [24]. Disadvantages of this technique include possible mass effect (which can lead to cerebral ischemia), increased risk of seizures, and higher esthetic impact. In order to avoid mass effect, a relatively wide bone window for temporal muscle insertion is recommended, as this allows to monitor cerebral hemodynamics alterations and perform early decompression and the revision of EMS as soon as the flow compromise is confirmed [25].

Surgical revascularization, including EMS, presents a substantial risk of cerebral ischemia due to the compression of the brain by temporal muscle swelling. In a recent paper, Macheda et al. elucidated that sagittal splitting of temporalis muscle might prevent the neurological deterioration caused by swollen temporalis muscle by reducing its volume without inhibiting the development of collateral vessels [26]. Matsushima et al. reported three refractory cases in which EDAS yielded to insufficient transdural anastomosis, and EMS was later performed in the posterior frontal and/or parietal regions of the same side with good results [16]. In 2017, enlarged encephaloduromyosynangiosis (EDMS) was first used in children. This technique provided better results in terms of compensation of the ACA territory when compared to standard EDMS [27].

In light of these limitations, Kinugasa et al. adopted an EDAMS approach (characterized by the combination of encephaloduroarteriosynangiosis (EDAS) and EMS) with the aim of maximizing the contact surface area with the brain, fostering the development of more anastomoses [10]. A further trick consisted in the use of a galea and periosteum ribbon to help the revascularization of medial frontal lobes [10]. Generally, all the techniques entailing the use of muscle carry the consequence of aesthetical problems. To address these disadvantages, Noguchi et al. reported a mixed series of patients treated with indirect revascularization techniques preserving the temporal muscle function through the exclusive use of the temporal fascia. With equal angiogenic effects as compared to standard procedures, this technique allowed to achieve good short- and mid-term cosmetic outcomes [28]. Yoshida et al. presented an EGMS technique that used a combination of temporal muscle, galea, and dura to form pedicles over the brain surface, preserving the STA [29]. The authors noticed that the procedure can be extended over a wider brain surface than the operative field and is suitable to establish a collateral circulation in the frontal lobe [29]. In addition, blood flow in the skin flap is maintained, with better aesthetical results [29].

### Donor tissue: periosteum, galea

#### EGPS-EGS-burr hole

The burr hole technique is an indirect revascularization procedure first described by Endo et al. in 1984 [30]. The procedure consists in drilling multiple holes through the skull to stimulate angiogenesis from the branches of the external carotid artery or middle meningeal artery (MMA) [30]. McLaughlin and Martin reviewed burr hole surgery in combination with either direct or indirect revascularization techniques as a single- or two-step procedure, reporting favorable clinical and angiographical outcomes [31]. Zhao et al. showed the advantages of multiple burr hole operation combined with dural inversion and periosteal synangiosis, reporting that sufficient neovascularization was achieved at 151 of the 160 burr holes [32]. The multiple burr hole operation combined with dural inversion and periosteal synangiosis is a simple, safe, and effective treatment for ischemic-type MMD, not requiring any other supplementary revascularization procedure. The extent of neovascularization includes not only the lateral surfaces of the hemispheres but also the medial surfaces (ACA territory) [32].

**Fig. 2:**
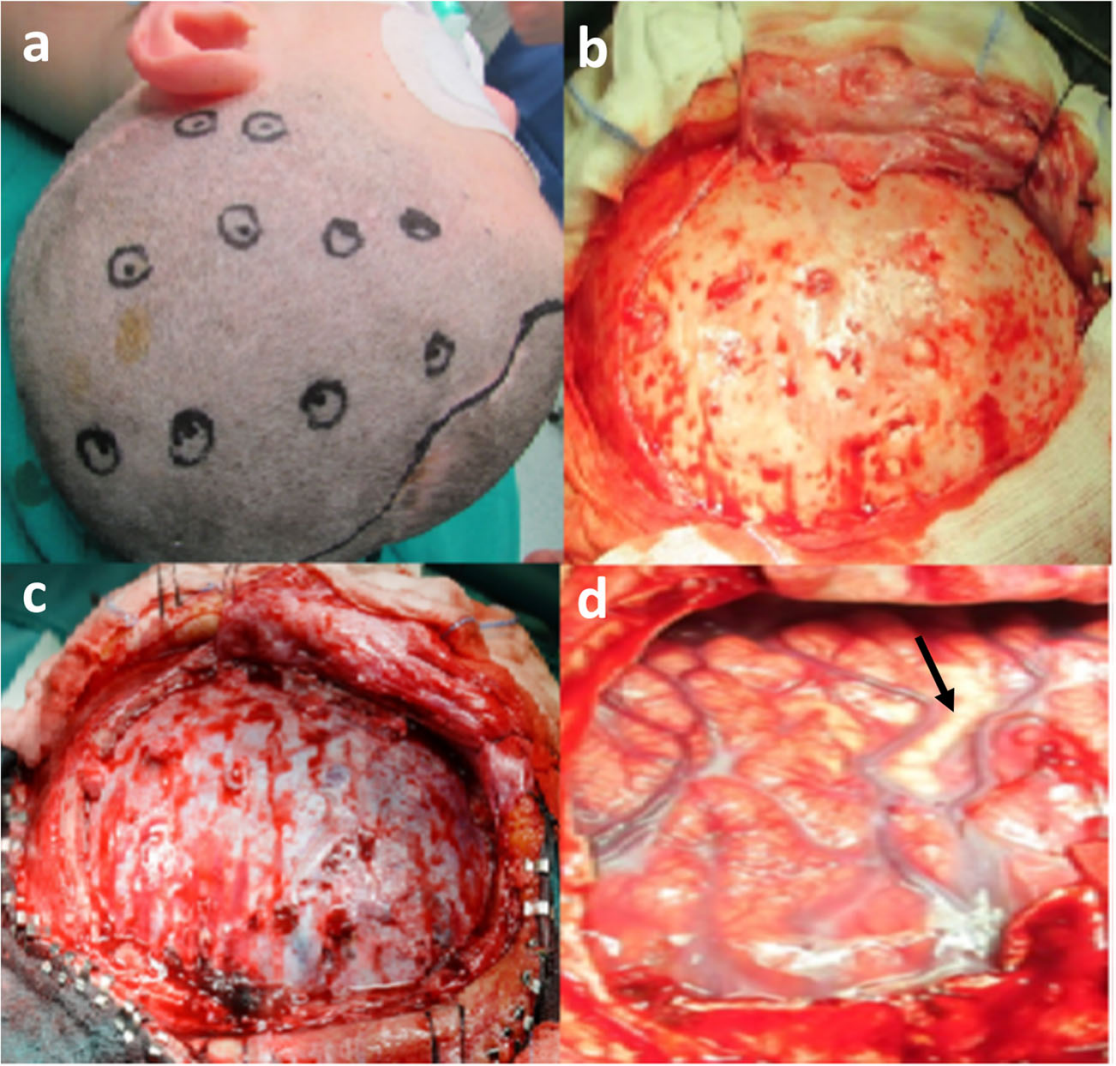
Illustration of burr hole complication. **a** Skin intraoperative burr holes mapping with neuronavigation system. **b** Closed or non-functional burr holes dissected in the periosteum flap. Preservation of effective holes at temporal base. **c** Residues of the dural route corresponding to the burr holes after craniotomy. **d** Cortical ischemia (black arrow) in a circumvolution corresponding to a non-functional hole

The disadvantages of multiple burr hole technique can be summarized as follows: (a) inability to support a homogeneous revascularization area; (b) inability to create angiogenesis if cortical subatrophy is present due to distance from brain surface; (c) the risk of early hole closure (Fig. 2); (d) difficulty to perform another indirect technique in case of failure; (e) risk of perioperative complications such as subdural effusions [33]. Pacetti et al. reported three cases of pediatric patients with MMS treated with multiple cranial burr holes that required a second surgical indirect revascularization due to radiological and clinical evidences of failure [34]. Scott et al. combined one frontal burr hole with pial synangiosis in MMS patients reporting good clinical results [35]. Ogiwara et al. considered preferable EDAS with bifrontal encephalogaleo(periosteal)synangiosis (EGS) in comparison to EGS alone to promote CBF in the ACA territory [36]. A hybrid technique is called encephalodurogaleosynangiosis (EDGS), in which the galea is placed over the medial cortex and into the interhemispheric fissure. However, the use of this procedure is limited due to the high risks of venous injury.

### Donor tissue: dura mater

#### Dural inversion technique and split DES

In 1997, Dauser et al. described the dural inversion technique, in which large dural flaps centered around the MMA are created to increase the cerebral surface coverage [37]. This procedure can be considered complimentary to other techniques. Gadgil et al. performed dural inversion combined with EDAS in 169 consecutive surgical procedures, showing a younger age of postoperative stroke and poor functional outcome [38]. Similar results were obtained by King et al. and McLaughlin et al. in both MMD and Quasi-MMD patients [31, 32]. Technically, if the STA is not incorporated, larger skin incision and craniotomy can be made in younger children, allowing to invert a larger dural surface area [31, 32]. Kashiwagi et al. successfully tested patients the combination of EDAS with split-duro-encephalo-synangiosis (split DES) in eighteen patients [39]. The dura surrounding the branches of the MMA was split into outer and inner layers, and the split surface of the outer layer was attached to the cortical surface improving the angiogenesis [39].

### Donor tissue: extracranial tissue

#### Omental transplantation

The intracranial omental transplantation is another non-anastomotic procedure. After its introduction by Karasawa in 1978, many case series on this technique have been published [10]. Omental transplantation requires both craniotomy and a large laparotomy, which is associated with relevant complications (fascial dehiscence, ventral hernia, and wound infection) in 18.5% of cases. In addition, there is a risk of intraoperative injury to the colonic vasculature present, especially in patients with history of previous surgery [40]. Further reported complications are hernia formation, bowel injury, ileus, bowel obstruction, peritonitis, and intestinal volvulus [40]. Of note, Navarro et al. emphasized that the use of laparoscopic technique permits to reduce the incision length, the bowel manipulation, and the rate of laparotomy-related complications [8].

### Limits and pitfalls of indirect revascularization

#### Overall evaluation of indirect procedures

The main goals of indirect bypass procedures are to decrease the incidence of future ischemic/hemorrhagic events and the long-term negative effects on cognitive functions in comparison to direct techniques (Table 1) [9]. However, the age of the patient and the pathophysiology of moyamoya vasculopathy can influence surgical results and revascularization time. Indeed, collateral pathways through indirect bypass do not develop in about 40–50% of adult patients and quasi-MM patients are known to be less prone to respond to this treatment [41–44]. A cohort study including 137 patients with moyamoya vasculopathy (MMD 74% with a mean age of 17.8 years; MMS 26% with a mean age of 10.3 years) treated with direct, indirect, and combined procedures showed no difference in postsurgical stroke risk between MMD and MMS (*p* = 0.787), confirming that affected individuals benefit equally from revascularization. In particular, a greater benefit of mainly indirect surgical revascularization was observed in cases of MMS [45]. A further criticism to the indirect techniques is represented by the latency between surgery and disease improvement. This delay is attributable to the time for vascular collaterals to develop, during which a perioperative ischemic stroke risk is present. Transdural anastomoses gradually develop 6 to 12 months after EDAS, in association with the decrease of moyamoya vessels and widening of STA and MMA [7, 46]. However, Matsushima et al. reported early clinical improvements (since the fourth postoperative day, mean 10 days) in 25 patients treated with EDAS [47]. Nearly half of these subjects (12 of 25) experienced an improvement of their neurological deficits or diminished attacks within 3 weeks from the procedure [47]. Eventually, a systematic review by Kazumata et al. showed that the postoperative stroke rate associated with direct or combined procedures was very similar to what observed after indirect procedures, supporting the safety of indirect techniques in MMD patients [48].

**Table 1:** Pros and cons of revascularization procedures and subtype classification

**Type of revascularization procedure**	**Advantages**	**Disadvantages**
Indirect	Suitable for young children and patients with absent/hypoplastic donor vessels; technically less demanding; shorter hospital stay; long-term and robust revascularization (potentially beyond the vascular territory); good postoperative neurological outcome	Slow revascularization; risk of stroke during the revascularization period
Direct	Early and robust revascularization	Technically demanding, especially in young children; dependent on the availability of donor vessels; longer hospital stay; limited revascularization outside the anastomosis; risk of hyperperfusion/reperfusion syndrome; risk of short- and long-term patency failure of anastomosis
Combined	Potential combination of the benefits of direct and indirect bypass; immediate and long-term revascularization; revascularization beyond the anastomotic area; more robust vascularization than bypass alone	Operative and perioperative disadvantages of direct bypass; time consuming; no significant difference/improvement in clinical outcome
**Major procedures**	**Additional procedures**	**Procedures limited to special cases**
EDAS	Burr holes, EMS	Double barrel (two branches, STA)
EMS	Burr holes, dural inversion	Split temporal muscle
EDAMPS	Dural inversion	
Pial synangiosis	Burr holes, EMS, GPS	Dural split
Burr holes	EDAS	
EGPS-EDGPS	Burr holes	Opening interhemispheric fissure

*Abbreviations*: *EDAS*, encephaloduroarteriosynangiosis; *EDAMPS*, encephaloduroarteriomyoperiosteosynangiosis; *EDGPS*, encephalodurogaleoperiosteal synangiosis; *EGPS*, encephalogaleoperiosteal synangiosis; *EMS*, encephalomyosynangiosis; *STA*, superficial temporal artery

#### Bypass effects in adult vs pediatric patients

In terms of outcome (5–20 years), the treatment with STA-MCA anastomosis and EDMAPS may lead to satisfactory outcomes in both pediatric and adult patients with moyamoya disease [42]. In particular, STA-MCA anastomosis and EDMAPS may lead to better long-term (> 5 years) outcome in pediatric patients than EDAS or STA-MCA anastomosis combined with EMS [42]. In a recent meta-analysis of 32 studies performed in Asian population (China and Japan), the incidence of cerebral hyperperfusion syndrome (CHS) was found to be lower in pediatric population (3.8%) than in adult patients (19.9%). No data are available on the comparison between the abovementioned procedures in these two groups. However, the main hypothesis is that the formation and development of collateral compensatory vessels in adults are generally less relevant than in children [42]. At the opening of the bypass, there is a sudden flow directed to the circulatory bed in the affected area, which is more difficult to tolerate in adults than in pediatric patients due to its extent. As a result, an increase in local perfusion with important neurological implications occurs [49]. Furthermore, no differences in CHS incidence between direct and combined procedures have been observed [49].

#### Indirect revascularization alone vs combined with bypass

The efficacy of encephaloduroarteriosynangiosis (EDAS) in adulthood-onset moyamoya disease (MMD) is well known. Dae Hee Han et al. compared the outcome of MMD patients treated with EDAS to those who did not receive any treatment [50]. The EDAS group had significantly better clinical outcomes than the untreated group after a median follow-up period of 12 months (*P* < 0.05) [50]. The authors concluded that the involvement of posterior circulation in MMD is not frequent and cerebral perfusion is preserved in adulthood-onset MMD patients [50]. This might explain why hemorrhages are frequent and there is a late onset of symptoms in adulthood-onset MMD [50]. As to the outcome in adult and pediatric patients, Ravindran et al. performed a meta-analytic study on 2258 pediatric patients receiving different indirect procedures (direct bypass, combined direct/indirect, and indirect bypass alone), with a mean follow-up duration of 71.4 ± 51.3 months [51]. Among these procedures, indirect consisted of 488 EDAS, 82 EDAMS, 410 EDAS+EGS, 216 pial synangiosis, and 107 EDAS+ dural inversion. The complication rates in patients treated with indirect revascularization and combined/direct bypass were comparable, being 15.4% and 9.0% respectively [51]. In adults, these rates have been reported to be 30.2% with direct bypass and 18.8% with indirect bypass, or 11% for both [52, 53]. In long-term outcomes of indirect bypass (EDAS for territory of MCA and bifrontal encephalogaleo(periosteal)synangiosis or multiple burr hole surgery was performed and combined with superficial temporal artery encephaloduroarteriosynangiosis for anterior circle, PCA territory (*n* = unilateral, 109 operations; bilateral, 49 operations)), encephaloduroarteriosynangiosis using the occipital artery or multiple burr hole surgery was performed for 629 children [54]. The results of incidence of late-onset ischemic stroke in this cohort was as low as 0.08% per year. After 6 months of surgery, there were few symptomatic infarctions on the operated hemisphere [54]. A crucial point is that indirect bypass is not sufficient alone to prevent recurrence of hemorrhage in pediatric patients, whereas it appears to be preventive in adults [54–56]. A comparative study on the efficacy of direct and indirect bypass surgery in terms of the postoperative risks and long-term effects in pediatric patients with ischemic-type MMD showed that the overall incidence of postoperative complications was not significantly different between the two groups (direct 17.6% vs. indirect 8.8%) [57]. The mean follow-up period was 71.9 ± 22.2 months for the direct bypass group and 60.2 ± 24.3 months for the indirect bypass group (*P* = 0.041) [57]. Kaplan-Meier analysis showed a longer stroke-free time in the direct bypass group than in the indirect bypass group (*P* = 0.025) [57]. Similarly, in some case series of adult patients, the postoperative ischemia rate ranged from 1.5 to 11.4% for direct/combined bypass [48, 58–60]. In a recent review by Zhao et al., the frequency of postoperative ischemia (5.5% for indirect bypass, 4.1% for direct bypass, and 5.8% for combined bypass) shows a uniform distribution of ischemic events after surgery regardless of the specific bypass type [61]. This is likely explained by the relationship between risk factors and postoperative ischemia due to hemodynamic brain compromise [61].

### Ischemic complications in EDAS

The post-EDAS increase in hemispheric and cortical flows have been reported to be significant in patients with TIAs, but not in those with cerebral infarctions [62]. According to Matsushima et al., these findings might be the consequence of technical difficulties and local factors influencing the cerebral blood circulation in the area where EDAS is performed [16]. Acute cerebral infarction following EDAS presents a distinctive territorial pattern, which is suggestive of an underlying occlusive mechanism [63]. No significant differences have been observed between the characteristics of postoperative infarctions after the first and the second stage of EDAS, suggesting that the operation stage does not directly affect the rate of postoperative infarction occurrence [63]. Furthermore, Kuroda et al. proposed that the progressive occlusion of the main cerebral arteries and the decreased anterior CBF resulting from the decreased moyamoya vessels represent the main factors underlying postoperative infarction in MMD patients showing frontal lobe infarction [64].

### Management of meningeal arteries

The MMA system is the dominant vascular supply to the ischemic hemisphere in the majority of moyamoya cases [2]. Accordingly, a procedure that incorporates a larger craniotomy with the preservation of the dural vasculature, wide arachnoidal dissection, and dural leaflet inversion will maximize the MMA contributions to the surgical revascularization, frequently exceeding the contribution provided by the STA [65]. On the basis of these suggested mechanisms, McLaughlin et al. developed a technique which uses dural splitting at the *locus minoris resistentiae* between the middle dural vascular layer and its internal median layer, and the application of the dural vascular layer to the brain surface after arachnoid opening. This technique was designed to optimize surgery for dural-pial synangiosis related to MMA branches [31].

#### Angiogenesis and surgical outcomes in pediatric and adult patients

Angiogenesis is a capillary sprouting triggered by hypoxia and resulting in higher capillary density, whereas arteriogenesis is a rapid proliferation of preexisting collateral arteries triggered by fluid shear stress leading to the formation of normal arteries [66]. One of the most relevant hypotheses is that functional revascularization requires two fundamental steps: the development of tissues providing vascular beds (angiogenesis) and the shear stress-related stimulus to induce arterious anastomosis (arteriogenesis) [67]. Accordingly, autoptic analysis on patients treated with indirect bypass showed poor revascularization if brain damage had occurred before the surgical procedure, which suggests that a reduced CPP manifests as a prolonged mean transit time (MTT) [68]. CPP influences the effects of indirect bypass surgery since arteriogenesis plays a pivotal role in the formation of the collateral network induced by the surgical procedure [68]. Once the connection between brain surface and external carotid artery system has been generated thanks to the indirect bypass procedure through scar formation and angiogenesis, arteriogenesis progresses from the external carotid artery system (normal CPP) to the brain surface (extremely decreased CPP) [68]. According to this interpretation of the effects of indirect bypass surgery, it is possible to predict the degree of arteriogenesis induced by surgical treatment [68]. Zhao et al. compared the revascularization degree on short-term (3–6 months ) and long-term (1 year) follow-up angiography in 25 hemispheres of patients with MMD (13 pediatric and 12 adult) treated with indirect bypass surgery (EDAS) to investigate the differences in the postsurgical neovascularization development in relation to the duration of the follow-up [57]. The global view in lateral DSA was not different in the two observation periods for both pediatric and adult patients. However, at 1-year follow-up, the number of collateral vessels had increased. Vein count is considered an indicator of the secondary effect of revascularization and is correlated with the level of Matsushima on follow-up. Interestingly, the process of formation of new venous vessels continues after 6 months from the surgical procedure and vein count is significantly higher at 1 year, although no significant difference was observed between pediatric and adult groups [57]. A comparative pediatric vs adult moyamoya ultrasound study further demonstrated that pediatric patients treated with STA and ECA might have a better postoperative increase in collateral vessels in comparison to adults [69]. Furthermore, pediatric patients in whom revascularization extended beyond the temporal region might have more collaterals than those in whom revascularization is limited to the temporal region [69]. In a case series of 75 patients treated with STA-MCA and EDMPS, Kuroda et al. observed that ischemic attacks in lower legs persisted in 56% of pediatric patients after EDAS or EMS and in 10% after STA-MCA anastomosis and EDAMS [42, 70]. The authors also showed that risk for perioperative complications is increased in combined surgical procedures due to longer operative times and wider surgical fields [42, 70].

### Skin incision

A relevant technical aspect in surgical revascularization procedures is represented by skin incision. Acker et al. analyzed different skin incisions and detected a lower overall wound complication rate in direct revascularization as compared to combined revascularization (3% vs 15.2%) [71]. Their observations emphasized that the complete Y incision may negatively impact wound healing in a significant way, leading to a higher rate of wound healing disorders in comparison to other type of skin incision (17.1% vs 3.1%) [71]. In order to mitigate these complications, Yokoyama et al. suggested three fundamental steps: (a) three-dimensional simulation imaging to confirm STA anatomy; (b) meticulous dissection of the STA on the epigaleal layer to protect the galeal layer; (c) careful consideration of the risk of scalp ischemia during any phase of surgery, until skin closure is completed [72]. The accurate adoption of these preventive measures can be advantageous to try to reduce skin incision–related complications in patients undergoing surgical revascularization.

### Pitfalls in anterior bypass surgery

The dynamical variations of the PCA circle taking place after an anterior bypass represent a relevant pathophysiological aspect in moyamoya vasculopathy treated with surgical revascularization. Steno-occlusive changes in the PCA have been reported in 26% of pediatric moyamoya patients [21]. The involvement of PCA in MMD is associated with the occurrence of neurological deficits before surgical treatment [21]. When PCA is affected, there is a higher prevalence of preoperative cerebral infarction and a more advanced stenosis of the ICA in comparison to cases in which other main vessels are primarily involved [22]. In particular, there is a 3-fold higher rate of perioperative stroke in pediatric patients with PCA involvement in comparison to cases where the PCA is not affected [22]. Kimiwada et al. reported a case series of pediatric patients younger than 16 years of age with progressive PCA stenosis after surgical anterior revascularization [21]. Of note, the occurrence of cerebral infarction and the younger age at the time of first diagnosis have been identified as the most relevant risks factor for the development of progressive PCA stenosis after anterior bypass surgery [21, 73].

## Conclusions

Indirect revascularization techniques represent a valuable therapeutic tool in the hand of the neurosurgeon to manage both MMS and MMS pediatric patients. The possible use of a large variety of tissues as blood supply increases the flexibility of these procedures, allowing to adopt the most appropriate technique on a case-by-case basis. Furthermore, indirect procedures may be used in combination with direct techniques, widening the spectrum of therapeutic resources in moyamoya patients. With regard to bypass procedures, a single best strategy does not exist as all techniques can be efficient in terms of protection against ischemic events in both pediatric and adult patients. However, the choice of the most appropriate technique has to be made based on the preoperative hemodynamic study (e.g., rapidity of the development of the stenosis), the age of the patient, and the preoperative neurological status. Indeed, all these factors can influence surgical outcome. Despite some undeniable limitations still exist and will need to be addressed in the next future, indirect techniques are currently an excellent alternative to direct revascularization, especially when a careful selection of the candidate patients is made.

## Data Availability

All data generated or analyzed during this study are included in this published article.
